# Pigs immunized with a novel E2 subunit vaccine are protected from subgenotype heterologous classical swine fever virus challenge

**DOI:** 10.1186/s12917-016-0823-4

**Published:** 2016-09-09

**Authors:** Rachel Madera, Wenjie Gong, Lihua Wang, Yulia Burakova, Karen Lleellish, Amy Galliher-Beckley, Jerome Nietfeld, Jamie Henningson, Kaimin Jia, Ping Li, Jianfa Bai, John Schlup, Scott McVey, Changchun Tu, Jishu Shi

**Affiliations:** 1 Department of Anatomy and Physiology, Kansas State University, Manhattan, KS 66506 USA; 2 Department of Diagnostic Medicine and Pathobiology, Kansas State University, Manhattan, KS 66506 USA; 3 Department of Chemistry, Kansas State University, Manhattan, KS 66506 USA; 4 Department of Chemical Engineering, Kansas State University, Manhattan, KS 66506 USA; 5 Institute of Military Veterinary Medicine, Academy of Military Medical Sciences, Changchun, China; 6 United States Department of Agriculture, Agricultural Research Service, Arthropod Borne Animal Disease Research Unit, Manhattan, KS 66502 USA

**Keywords:** Classical swine fever, Vaccine, E2, Adjuvant, CSF, CSFV, KNB-E2

## Abstract

**Background:**

Classical swine fever (CSF) or hog cholera is a highly contagious swine viral disease. CSF endemic countries have to use routine vaccination with modified live virus (MLV) vaccines to prevent and control CSF. However, it is impossible to serologically differentiate MLV vaccinated pigs from those infected with CSF virus (CSFV). The aim of this study is to develop a one-dose E2-subunit vaccine that can provide protection against CSFV challenge. We hypothesize that a vaccine consisting of a suitable adjuvant and recombinant E2 with natural conformation may induce a similar level of protection as the MLV vaccine.

**Results:**

Our experimental vaccine KNB-E2 was formulated with the recombinant E2 protein (Genotype 1.1) expressed by insect cells and an oil-in-water emulsion based adjuvant. 10 pigs (3 weeks old, 5 pigs/group) were immunized intramuscularly with one dose or two doses (3 weeks apart) KNB-E2, and 10 more control pigs were administered normal saline solution only. Two weeks after the second vaccination, all KNB-E2 vaccinated pigs and 5 control pigs were challenged with 5 × 10^5^ TCID_50_ CSFV Honduras/1997 (Genotype 1.3, 1 ml intramuscular, 1 ml intranasal). It was found that while control pigs infected with CSFV stopped growing and developed high fever (>40 °C), high level CSFV load in blood and nasal fluid, and severe leukopenia 3–14 days post challenge, all KNB-E2 vaccinated pigs continued to grow as control pigs without CSFV exposure, did not show any fever, had low or undetectable level of CSFV in blood and nasal fluid. At the time of CSFV challenge, only pigs immunized with KNB-E2 developed high levels of E2-specific antibodies and anti-CSFV neutralizing antibodies.

**Conclusions:**

Our studies provide direct evidence that pigs immunized with one dose KNB-E2 can be protected clinically from CSFV challenge. This protection is likely mediated by high levels of E2-specific and anti-CSFV neutralizing antibodies.

## Background

Classical swine fever (CSF) or hog cholera, causes severe economic losses to the swine industry worldwide and presents a significant agro-security threat to CSF free countries such as the U.S. CSF is a highly contagious viral disease of swine, including wild (feral) pigs. CSF is caused by an enveloped Pestivirus named classical swine fever virus (CSFV) [1]. The CSFV genome consists of a single, positive-stranded RNA of approximately 12.3 kb encoding a polyprotein of 3898 amino acids. The translated polyprotein is processed by viral as well as cellular proteases to the mature forms of four structural (C, E^rns^, E1, and E2) and eight nonstructural viral proteins (N^pro^, p7, NS2, NS3, NS4A, NS4B, NS5A, and NS5B) [2].

The genotypes of CSF viruses can be classified into three major groups with eleven subgroups [3–5]. Group 1 (1.1, 1.2, 1.3, & 1.4) contains primarily historical strains isolated from many regions of the world and includes all live-attenuated vaccine strains. Group 2 (2.1, 2.2, & 2.3) contains most of the currently circulating strains, whose prevalence has increased and caused epidemic infection since the 1980s. Group 3 (3.1, 3.2, 3.3, & 3.4) contains most of the strains distributed in separated geographic regions such as Taiwan, Korea, Japan, Thailand and the United Kingdom. Recent phylogenetic analyses have indicated a “switch” of field CSFV from the historical group 1 or 3 to the more recently prevalent group 2 in Europe and Asia [6, 7].

In contrast to the non-vaccination and stamping-out policy in CSF-free zones, CSF endemic countries have to use routine vaccination to prevent and control CSF. When used properly, vaccination can be an effective approach to limit transmission of the CSFV, prevent disease outbreaks, and establish protective immunity in naïve pig populations. To date, three types of CSF vaccines have been developed commercially: 1) modified live virus (MLV) vaccines which are manufactured and widely used in CSF endemic countries [8, 9]; 2) subunit vaccines based on CSF viral envelope protein E2 [10–13] , and 3) a chimeric live recombinant viral vector vaccine [14–17]. Although they are important tools for CSF outbreak control, better CSF vaccines are needed for the U.S. to maintain the CSF-free status because of the intrinsic limitations of the current commercial vaccines.

MLV CSF vaccines are generally safe and effective. However, it is impossible to serologically differentiate MLV vaccinated pigs from those infected with CSFV. Thus, a long and costly non-vaccination and stamping-out eradication process would have to be followed if MLV are the only vaccines available. In addition, it would be unsafe and costly to manufacture MLV vaccines in CSF-free countries such as the U.S. In 2015, Suvaxyn CSF Marker, a chimeric CSF vaccine that contains live bovine viral diarrhea virus (BVDV) which has been modified to express CSF E2 gene was approved by the European Union. However, Suvaxyn CSF Marker is approved only for emergency vaccination (page 6 in SUMMARY OF PRODUCT CHARACTERISTICS, http://ec.europa.eu/health/documents/community-register/2016/20160704135310/anx_135310_en.pdf).

Compared to MLV vaccines, subunit vaccines are designed to meet the DIVA (differentiation of infected from vaccinated animals) requirement for vaccination. Two CSFV envelope glycoproteins E^rns^ and E2 have been targeted for vaccine development. Although the effectiveness of E^rns^ as a vaccine target has been controversial [18, 19], two vaccines (*BAYOVAC CSF E2* from Bayer and *Porcilis Pesti* from MSD) based on baculovirus-expressed E2 were marketed commercially in Europe. Vaccinated pigs develop antibodies exclusively to the E2 protein; whereas, naturally infected animals may also develop antibodies to E^rns^, thus permitting detection of vaccinated animals via this negative marker [20].

However, these subunit vaccines are no longer commercially available because of two significant weaknesses compared with conventional MLV CSF vaccines: they need two vaccinations and offer incomplete protection. In addition to insect cells, yeast and mammalian cells are also used to produce E2 antigens for vaccine development [18, 21]. However, two vaccinations are also required for these yeast- or mammalian cell-based E2 subunit vaccines to achieve homologous protection in pigs. Despite the limitations of E2-subunit vaccines, E2 protein is well recognized as the protective antigen that is essential and may be sufficient for vaccine-mediated protection against CSFV.

One major objective of our CSF research is to develop a DIVA CSF vaccine that can be safely manufactured and used in the U.S. We have recently found that the monoclonal anti-E2 antibody WH211 has much stronger affinity to the dimeric E2 than the monomer. Others have recently shown that antibodies specific to one genotype E2 might not have strong affinity to other genotype E2 proteins on CSFV [22], and this may partially explain why limited protection against heterologous CSFV occurred in pigs vaccinated with E2-subunit vaccines in which E2-specific antibodies play an important role in protective immunity. In addition, we have recently demonstrated that adjuvants can enhance vaccine-mediated cross-protection against porcine reproductive and respiratory syndrome virus (PRRSV) [23] and swine influenza virus [24]. Thus, we hypothesize that a vaccine consisting of a suitable adjuvant and recombinant E2 with natural conformation from the C-strain may induce similar levels of protection as MLV CSF vaccines. Here we provide the first evidence that pigs immunized with a novel one-dose E2-subunit vaccine (KNB-E2) are protected clinically from CSFV challenge. This protection is likely mediated by high levels of E2-specific anti-CSFV neutralizing antibodies.

## Methods

### Virus and cells

Classical swine fever virus isolate Honduras/1997 (a field isolate from Honduras) was kindly provided by Dr. Sabrina Swenson from the Animal and Plant Health Inspection Service (APHIS), United States Department of Agriculture (USDA). This CSFV isolate was passaged four times in swine testicle cells (ST; ATCC) cultured in DMEM (Gibco) supplemented with 10 % fetal bovine serum (FBS; Atlanta Biologicals) and 1 % Penicillin-streptomycin solution (Gibco). For recombinant E2 production in insect cells, *Spodoptera frugiperda* insect cells (Sf9; ATCC) were grown in Grace’s insect medium (Gibco) supplemented with 10 % FBS and 1 % antibiotic-antimycotic solution (Gibco), and High Five insect cells (Invitrogen) were grown in Express Five SFM medium (Gibco).

### Expression and purification of recombinant CSFV E2 and E^rns^ protein

PCR-amplified CSFV E2 and E^rns^ genes from hog cholera lapinised virus C-strain (HCLV, Genotype 1.1) was cloned into pFastBacTMI Baculovirus Expression System plasmid vector using the following primers: HCLV-E2-F: 5′-CGCGGATCCACCATAACCATTGCATTCCTCATC-3′, HCLV-E2-R: 5′-CCGGAATTCTTAAT-GATGGTGATGATGCGCATCCAGGTCAAACCAG-3′; HCLV-E^rns^-F: 5′-CGCGGATCCACCATGGAAAAAGCCCTATT-GGCATG-3′, and HCLV-E^rns^-R: 5′-CCGGAATTCTTAATGGTGATGGTGATGATGCACCCTCGCTGCTCCCTGTC-3′. The desired PCR products were then transformed into DH10BacTM *E. coli* host strain that contains a baculovirus shuttle vector (bacmid) and a helper plasmid. Upon screening of colonies, positive *E. coli* transformants with recombinant E2 and E^rns^ bacmid were upscaled by overnight culture in liquid media and the bacmid was isolated using Purelink® HiPure Plasmid Midiprep Kit (Invitrogen). To generate recombinant baculovirus stock for E2 and E^rns^ expression, Sf9 insect cells were transfected using Cellfectin II Reagent (Invitrogen) and passaged three times to amplify the E2- or E^rns^ bearing recombinant baculovirus. At passage 3, Sf9 cell culture supernatant was collected and clarified by centrifugation at 500 × *g* for 5 min to obtain the baculovirus stock that was used to infect High Five™ Cells for E2 or E^rns^ expression. E2 and E^rns^ protein was purified using Ni-NTA Agarose (Novex™) as described by the manufacturer. CSFV E2 protein expression and purification was verified by SDS-PAGE and subsequently by western blot using E2 monoclonal antibody WH211 (APHA Scientific) as we described previously [25].

### Pigs, E2-subunit vaccine, vaccination, and challenge

Conventional Large White-Duroc crossbred weaned specific-pathogen free male piglets (3 weeks of age) were purchased from a commercial vendor. The pigs were fed with standard commercial diet and kept under laboratory biosafety level III Agriculture (BSL3-Ag) conditions at the Biosecurity Research Institute (BRI), Kansas State University.

The CSFV E2 subunit vaccine KNB-E2 was prepared by simple hand mixing of purified CSFV E2 with an oil-in-water emulsion adjuvant [24]. One dose (2 ml) KNB-E2 contains 75 μg of purified E2 protein. The pigs were randomly allotted into 4 groups (*n* = 5 for each group) with two control groups and two vaccinated groups. The control groups including non-vaccinated, non-challenged (−/−) and non-vaccinated, CSFV challenged (−/+) pigs were given intramuscularly 2 ml Phosphate-buffered saline (PBS). All vaccinated pigs were immunized intramuscularly with 2 ml of KNB-E2. One vaccinated group received only one dose of the KNB-E2 (One-dose group) and the second vaccinated group received two doses of KNB-E2 with the second dose given 21 days later (Two-dose group). Two weeks after the second vaccination, pigs were challenged with 5 × 10^5^ TCID_50_ CSFV isolate Honduras/1997 (1 ml intramuscular, 1 ml intranasal). Honduras/1997 was evaluated as a moderate virulent strain in our previous study. E2 sequencing and nucleotide BLAST analysis indicate that this isolate belongs to CSFV subgenotype 1.3 (GenBank Accession#: KU716076) and has 97 % nucleotide sequence identity to a CSFV isolated in nearby Guatemala (Accession # JX028200).

Pigs were monitored daily for clinical signs and rectal temperatures. Sera were collected on study day 0 (Dose 1) and 21 days post first vaccination (21 DPV; Dose 2). Whole blood, serum and nasal swabs were collected on 35 DPV (also as 0 DPC) and every 3 days after challenge until the end of this study, at 15 days post challenge (15 DPC). All pigs that survived were humanely euthanized at 15 DPC. Total white blood cell (WBC) and leukocyte differentiation counts were performed with VetScan HM5 Analyzer (Abaxis). Tonsil, lymph node, kidney, and lung were collected for virus identification by immunohistochemical staining with anti-E2 antibody WH303 as described earlier with some modification [26].

### Measurement of anti-E2 and anti-E^rns^ antibodies in pigs

Anti-E2 and anti-E^rns^ antibodies were determined in E2-vaccinated and CSFV-infected pig sera by enzyme-linked immunosorbent assay (ELISA). Briefly, 62.5 ng/ml of purified E2 or E^rns^ was used as coating antigen on 96-well flat-bottomed microtiter plates (Corning®). Diluted sera (each sample in duplicate) were added to plates and incubated for 1 h at room temperature. Then, horseradishperoxidase (HRP)-conjugated goat anti-porcine IgG was used as secondary antibody (Southern Biotech). The ELISA plates were developed using 3,3,5,5 tetramethylbenzidine (TMB) stabilized chromogen (Novex™), and the reactions were stopped with 2 N sulfuric acid. Relative antibody concentrations were determined using optical spectrophotometer readings at 450 nm using a SpectraMAX microplate reader and analyzed with Softmax® Pro 6.4 Software (Molecular Devices).

### RNA isolation and real-time RT-PCR for virus quantification in sera and nasal swabs

Viral RNA from sera and nasal swabs were isolated using IBI Viral Nucleic Acid Extraction Kit II (IBI Scientific) as prescribed by the manufacturer. Real-time RT-PCR was performed using CSFV-specific primers and PCR cycling parameters as previously described [27]. For quantification, passage 4 CSFV stock (10^7^ TCID_50_) was serially diluted (10^7^ to 10^2^) before viral RNA isolation and used for standard curve. Viremia was calculated and determined using StepOne™ Software v2.3 (Applied Biosystems).

### Serum anti-CSFV neutralization assay

The anti-CSFV neutralizing antibody titers in the serum were determined using indirect fluorescent antibody assay (IFA). Briefly, serum samples collected at 35 DPV (0 DPC) and 15 DPC were first diluted five-fold and then serially diluted two-fold, the diluted serum samples (in duplicate) were incubated with 100 TCID_50_ of CSFV Honduras/1997 in DMEM with 10 % FBS for 1 h at 37 °C. Residual virus infectivity was determined by adding 1.0 × 10^4^ ST cells to each well with serum-virus mixture in 96-well plate and incubated at 37 °C for 3 days. The cells were subjected to immunofluorescence staining with E2-specific mAb WH211 and Alexa Fluor 488 goat anti-mouse IgG (H + L) (Life Science). Neutralizing antibody titers in serum samples were expressed as the reciprocal of the highest dilution that caused 50 % neutralization.

### Statistical analysis

The variations between groups were analyzed by one-way analysis of variance (ANOVA) followed by post-hoc Dunnett’s method using Sigmaplot 11 software (Systat). Differences were considered statistically significant when *p* < 0.05.

## Results

### Recombinant CSFV E2 proteins produced by insect cells existed as homodimers and had stronger affinity to anti-E2 antibody than that of the monomeric E2 proteins

**Fig. 1:**
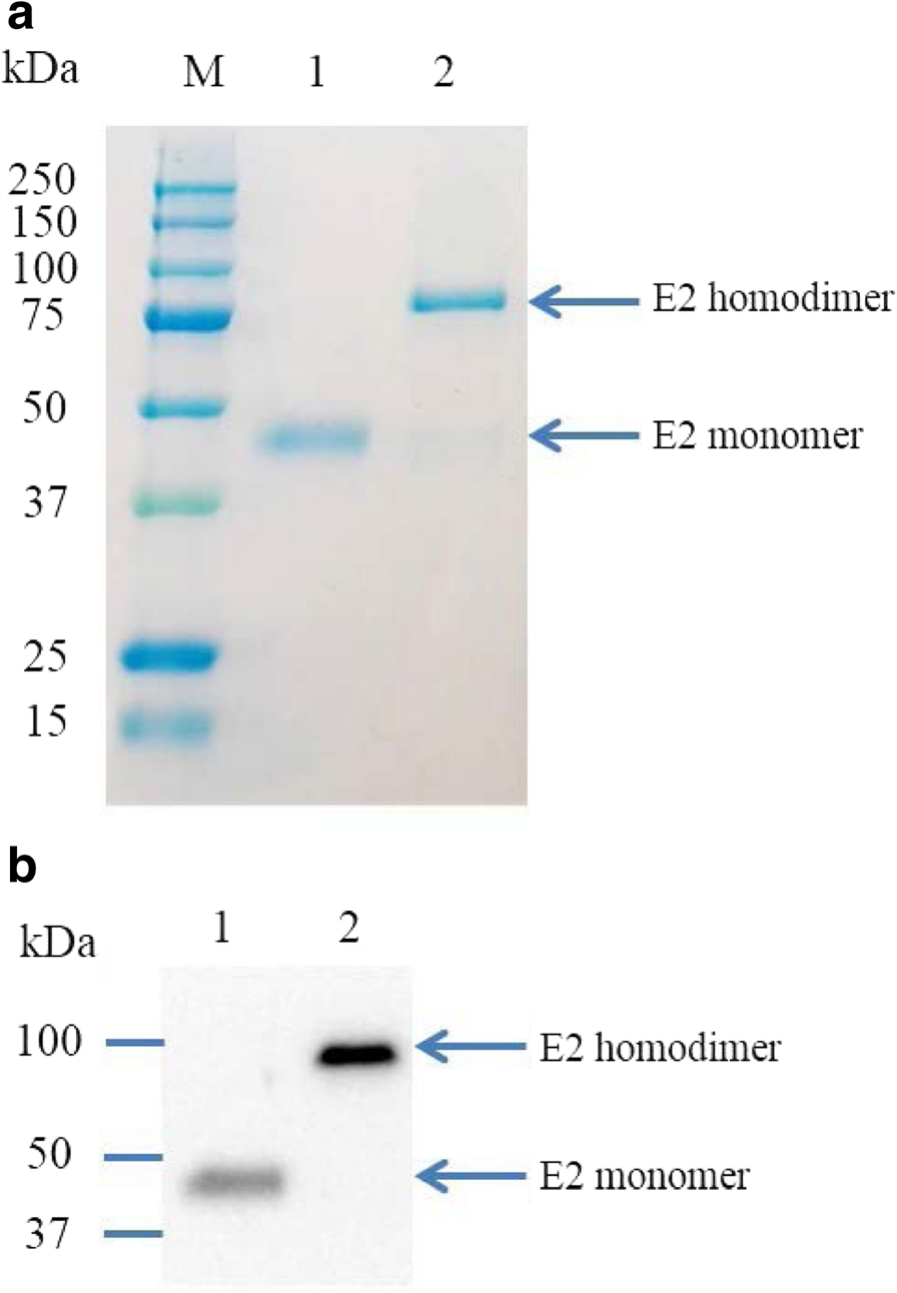
Production and characterization of recombinant CSFV E2 protein. **a** SDS-PAGE analysis. Lane 1, E2 protein (1 μg) treated with Laemmli sample buffer with addition of reducing reagent β-mercaptoethanol (β-ME); Lane 2, E2 protein (1 μg) treated with Laemmli sample buffer without β-ME. **b** Western blot of purified E2 protein. Lane 1, E2 protein (1 μg) treated with β-ME; Lane 2, E2 protein (50 ng) purified and stored under non-reducing conditions. E2-specific Mab WH211 was used for the western blot

CSF virus E2 gene from the HCLV was cloned into the recombinant baculovirus and recombinant E2 protein was produced using High Five insect cells. The culture medium was then collected by centrifugation and subjected to protein purification with Ni-NTA agarose beads. As shown in Fig. 1a, pure recombinant E2 protein was obtained from the condition medium. Under reducing conditions, recombinant E2 protein appeared to be 45 kDa, while the native E2 protein mainly existed as homodimer under non-reducing conditions with a molecular weight of ~90 kDa (Fig. 1a). The dimerization of recombinant E2 protein expressed in insect cells was further confirmed by western blot analysis with anti-E2 mAb WH211. It is worth noting that although the amount of E2 dimer protein loaded in lane 2 on Fig. 1b was only 1/20 of the monomeric E2 (50 ng vs. 1 μg), E2 dimers seem to have a higher affinity to WH211 than did the E2 monomer as evidenced by the difference in intensity of the E2 bands on western blot (Fig. 1b).

### Pigs immunized with one dose KNB-E2 were protected from heterologous CSFV challenge

**Fig. 2:**
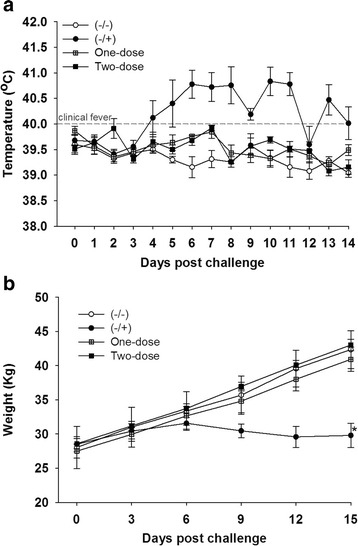
Pigs immunized with E2 subunit vaccine KNB-E2 were protected clinically from CSFV challenge. Pigs were immunized with KNB-E2 on Day 0 for the One-dose group and a second dose on 21 DPV for the Two-dose group. Two weeks after the second vaccination (35 DPV), pigs were challenged with 5 × 10^5^ TCID_50_ CSFV strain Honduras/1997. **a** Body temperature was monitored daily after CSFV infection. Vaccinated pigs did not have body temperatures higher than 40.5 °C. **b** Shown are body weight measured every 3 days after CSFV challenge. Data are mean ± SEM for five pigs per group. * *p* < 0.05

**Fig. 3:**
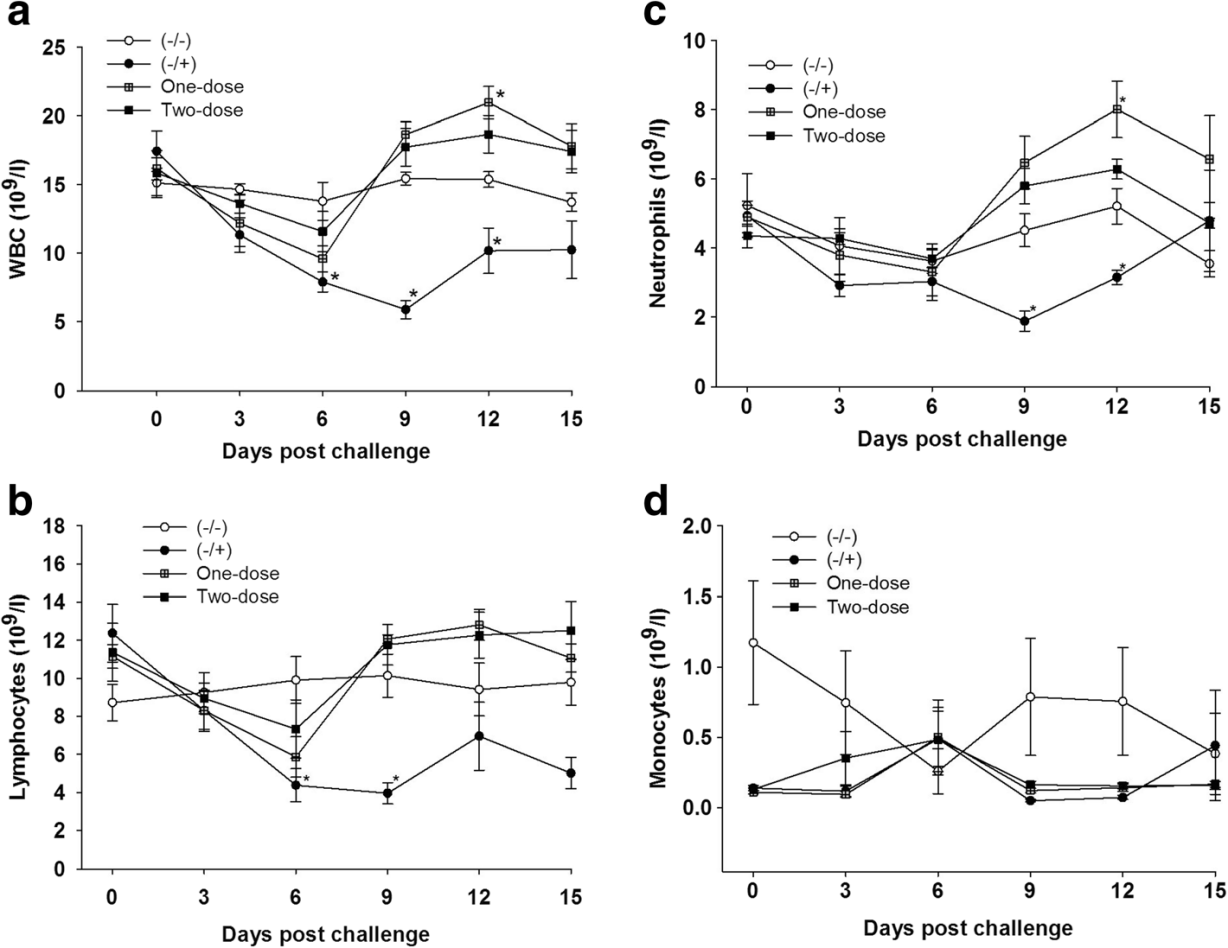
Pigs vaccinated with KNB-E2 were protected from CSFV-induced leukopenia. Blood cell counts including WBC (**a**), lymphocytes (**b**), neutrophils (**c**), and monocytes (**d**) were monitored on 0, 3, 6, 9, 12, and 15 DPC. A slight decrease but not statistically different in the numbers of WBC, lymphocytes, and neutrophils of all vaccinated pigs were observed at 3 DPC and 6 DPC, these numbers were recovered by 9 DPC. Data are mean ± SEM for five pigs per group. *p < 0.05

To test the efficacy of CSFV E2 subunit vaccine, pigs in both vaccinated groups and the (−/+) group were challenged with CSFV isolate Honduras/1997. After CSFV inoculation, pigs in the (−/+) group displayed clinical signs of CSF, including high fever (Fig. 2a), loss of body weight (Fig. 2b), severe leukopenia (Fig. 3), convulsion, diarrhea, and one of the five pigs, #59, had to be euthanized due to severe clinical symptoms at 9 DPC. In contrast, pigs vaccinated with KNB-E2 - the E2 subunit vaccine (One-dose and Two-dose groups) did not show elevated body temperature after CSFV challenge (Fig. 2a). More importantly, body weight gains in the two vaccinated groups were almost identical to that in healthy control pigs (−/−) before and after CSFV challenge (Fig. 2b).

WBC and lymphocyte counts in (−/+) pigs challenged with CSFV were significantly reduced after CSFV inoculation and reached the lowest levels at 9 DPC (Fig. 3a and b), respectively. In contrast, there was a slight decrease in the numbers of WBC and lymphocytes in pigs vaccinated with KNB-E2 at 3 and 6 DPC; however, the numbers of these cells in vaccinated pigs, especially for pigs in the One-dose group, increased significantly at 9 DPC and then after (Fig. 3a and b, respectively). Similarly, the numbers of neutrophils in the (−/+) group were significantly reduced at 9 and 12 DPC (Fig. 3c), while the numbers of neutrophils in pigs immunized with KNB-E2 were increased significantly at 9 and 12 DPC. The numbers of monocytes in all pig groups seem not to be affected significantly by the CSFV challenge (Fig. 3d).

### Vaccination prevents CSF virus replication in blood, nasal cavity, lung, lymph node, tonsil, and kidney in pigs immunized with KNB-E2

CSFV was detected in both serum and nasal swab samples from the (−/+) pigs at 6 DPC. The virus loads in these pigs continued to increase at 9 and 12 DPC and reduced slightly at the time of necropsy on 15 DPC (Tables 1 and 2). In contrast, pigs vaccinated KNB-E2 (One-dose or Two-dose) had undetectable or extremely low levels of CSFV RNA in the blood and nasal fluids by real-time RT-PCR analysis (Tables 1 and 2). CSFV was not detected in any of the serum samples from KNB-E2 vaccinated pigs when they were added to ST cells (10 μl/well in a 96-well plate) for virus isolation (data not shown); and using the same method, CSFV was detected in serum samples from (−/+) pigs collected on 6 and 9 DPC (data not shown). Surprisingly, one of the (−/+) pigs, #56, had significantly less CSFV in the blood and nasal fluid than that of other pigs in the (−/+) group.

**Table 1:** Pigs vaccinated with E2 subunit vaccine did not develop viremia after CSFV challenge

Serum		3 DPC		6 DPC		9 DPC		12 DPC		15 DPC	
Treatment	Pig #	Ct value	TCID_50_	Ct value	TCID_50_	Ct value	TCID_50_	Ct value	TCID_50_	Ct value	TCID_50_
(−/−)	51	(−)	(−)	ND	ND	ND	ND	ND	ND	ND	ND
(−/−)	52	37	98	ND	ND	ND	ND	(−)	(−)	(−)	(−)
(−/−)	53	(−)	(−)	(−)	(−)	ND	ND	(−)	(−)	36	112
(−/−)	54	(−)	(−)	(−)	(−)	(−)	(−)	(−)	(−)	(−)	(−)
(−/−)	55	(−)	(−)	(−)	(−)	(−)	(−)	(−)	(−)	(−)	(−)
(−/+)	56	(−)	(−)	29	4330	30	2774	28	17,617	35	163
(−/+)	57	36	78	25	47,547	20	1,460,325	19	4,993,009	21	1,036,940
(−/+)	58	(−)	(−)	28	7090	23	222,328	20	2,587,042	21	1,095,457
(−/+)	59	(−)	(−)	26	28,553	22	480,384	pig died			
(−/+)	60	(−)	(−)	29	5169	22	507,102	21	1,019,529	21	1,003,013
One-dose	61	37	42	28	7192	35	160	38	29	38	58
One-dose	62	(−)	(−)	34	170	(−)	(−)	39	21	(−)	(−)
One-dose	63	34	179	35	160	36	68	(−)	(−)	(−)	(−)
One-dose	64	36	56	33	330	34	169	36	131	(−)	(−)
One-dose	65	(−)	(−)	35	100	(−)	(−)	(−)	(−)	(−)	(−)
Two-dose	66	(−)	(−)	36	56	35	106	(−)	(−)	(−)	(−)
Two-dose	67	(−)	(−)	34	279	(−)	(−)	(−)	(−)	(−)	(−)
Two-dose	68	(−)	(−)	36	85	(−)	(−)	(−)	(−)	(−)	(−)
Two-dose	69	(−)	(−)	(−)	(−)	(−)	(−)	(−)	(−)	(−)	(−)
Two-dose	70	(−)	(−)	35	97	(−)	(−)	(−)	(−)	(−)	(−)

*ND* not done; (−): Undetectable; Pigs were challenged with CSFV 35 days post first vaccination. CSFV RNA in the blood was measured by real-time RT-PCR as described in Methods section

(−/−): Control pigs without CSFV challenge; (−/+): Control pigs challenged with CSFV

One-dose: Pigs vaccinated with one dose KNB-E2 and then challenged with CSFV

Two-dose: Pigs vaccinated with KNB-E2 twice and then challenged with CSFV

**Table 2:** CSFV were cleared from the nasal cavity in pigs vaccinated with E2 subunit vaccine 15 days post challenge

Nasal swab		3 DPC		6 DPC		9 DPC		12 DPC		15 DPC	
Treatment	Pig #	Ct value	TCID_50_	Ct value	TCID_50_	Ct value	TCID_50_	Ct value	TCID_50_	Ct value	TCID_50_
(−/−)	51	ND	ND	ND	ND	ND	ND	ND	ND	(−)	(−)
(−/−)	52	ND	ND	ND	ND	ND	ND	(−)	(−)	(−)	(−)
(−/−)	53	(−)	(−)	(−)	(−)	(−)	(−)	(−)	(−)	(−)	(−)
(−/−)	54	(−)	(−)	(−)	(−)	39	37	(−)	(−)	(−)	(−)
(−/−)	55	(−)	(−)	34	184	34	560	(−)	(−)	(−)	(−)
(−/+)	56	(−)	(−)	35	95	33	1521	28	19,033	33	2867
(−/+)	57	(−)	(−)	32	876	24	300,220	20	1,882,476	22	904,988
(−/+)	58	37	31	34	293	24	411,326	21	1,438,113	21	1,502,256
(−/+)	59	(−)	(−)	32	1065	21	3,182,081	pig died			
(−/+)	60	38	19	34	261	25	293,326	21	1,451,296	22	745,304
One-dose	61	(−)	(−)	35	124	36	166	36	106	(−)	(−)
One-dose	62	(−)	(−)	(−)	(−)	38	52	37	42	36	109
One-dose	63	(−)	(−)	37	35	37	83	33	495	(−)	(−)
One-dose	64	(−)	(−)	34	224	(−)	(−)	37	68	(−)	(−)
One-dose	65	(−)	(−)	36	68	37	78	34	301	(−)	(−)
Two-dose	66	(−)	(−)	37	38	39	28	35	145	(−)	(−)
Two-dose	67	(−)	(−)	36	60	38	67	35	221	(−)	(−)
Two-dose	68	(−)	(−)	(−)	(−)	34	2398	34	283	(−)	(−)
Two-dose	69	(−)	(−)	36	56	37	79	34	269	37	42
Two-dose	70	(−)	(−)	36	58	(−)	(−)	34	330	(−)	(−)

*ND* not done; (−): Undetectable; Pigs were challenged with CSFV 35 days post first vaccination. CSFV RNA in the blood was measured by real-time RT-PCR as described in Methods section

(−/−): Control pigs without CSFV challenge; (−/+): Control pigs challenged with CSFV

One-dose: Pigs vaccinated with one dose KNB-E2 and then challenged with CSFV

Two-dose: Pigs vaccinated with KNB-E2 twice and then challenged with CSFV

Using immunohistochemical staining with E2-specifc antibody WH303, we also determined whether CSFV were present in the lung, lymph nodes, kidney, and tonsil collected from all groups at necropsy (15 DPC). It was found that CSFV were present in the tonsil, lymph node, lung, and kidney in 4/5 of the (−/+) pigs (data not shown). Consistent with the real-time RT-PCR data shown above, CSFV was not detected in any of the tissues from pig #56. CSFV was not detected in any tissues from pigs vaccinated with KNB-E2 (One-dose or Two-dose).

### Pigs vaccinated with KNB-E2 developed high levels of E2-specific antibodies and anti-CSFV neutralizing antibody (VNA) titers after vaccination and challenge

**Fig. 4:**
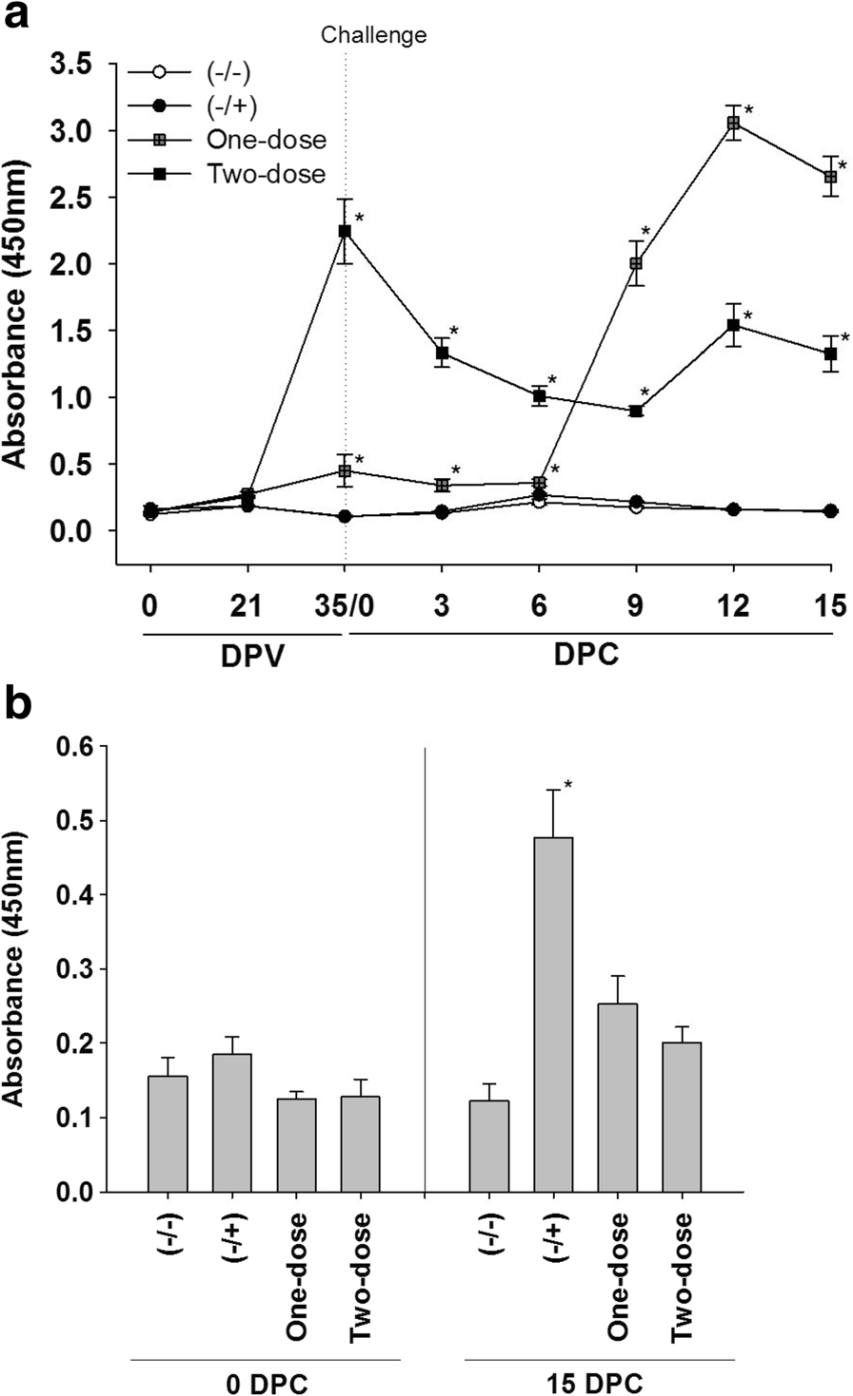
E2-specific antibodies were detected by ELISA only in pigs vaccinated with KNB-E2 before and after challenge. E2- and E^rns^-specific antibodies were measured by ELISA as we described in Materials and Methods. **a** E2-specific antibody in serum samples collected after vaccination and challenge. **b** E^rns^-specific antibody in serum samples collected at 0 DPC and 15 DPC. Data are shown as mean ± SEM for five pigs per group. * *p* < 0.05

As shown in Fig. 4a, all vaccinated pigs developed E2-specific antibody after immunization. E2-specific antibody level in the One-dose group increased dramatically after challenge and was even higher than that in the Two-dose group at 9 DPC. The level of E2-specific antibody in the Two-dose group increased dramatically after the boost vaccination, but decreased significantly in the first 9 days post challenge. E2-specific antibody was not detected in control pigs before or after the challenge (Fig. 4a). In contrast to E2-spepcific antibody response, E^rns^-specific antibody was only detected in the (−/+) pigs at 15 DPC (Fig. 4b).

Similar to E2-specific antibody response, all vaccinated pigs developed anti-CSFV neutralizing antibody before challenge at 35 DPV (Table 3). The VNA titers in the One-dose group were lower than that in the Two-dose group at 35 DPV. However, the VNA titers in the One-dose group were higher than that in the Two-dose group at 15 DPC. Pigs in the control groups had no detectable neutralizing antibody at 35 DPV and 15 DPC. Thus, there seems to be close correlation between anti-CSFV neutralizing antibody titers and levels of E2-specific antibodies in pigs after KNB-E2 vaccination and CSFV challenge. Consistent with its viremia status, pig #56 in the (−/+) group had detectable anti-CSFV neutralizing antibody at 15 DPC.

**Table 3:** Pigs vaccinated with E2 subunit vaccine developed high titers of anti-CSFV neutralizing antibodies

Treatment	Pig #	35 DPV	15 DPC	Treatment	Pig #	35 DPV	15 DPC
(−/−)	51	0	0	(−/+)	56	0	>160
(−/−)	52	0	0	(−/+)	57	0	0
(−/−)	53	0	0	(−/+)	58	0	0
(−/−)	54	0	0	(−/+)	59	0	0
(−/−)	55	0	0	(−/+)	60	0	0
One-dose	61	15	10,240	Two-dose	66	960	7680
One-dose	62	240	>10,240	Two-dose	67	640	7680
One-dose	63	320	>10,240	Two-dose	68	1920	10,240
One-dose	64	20	>10,240	Two-dose	69	7680	7680
One-dose	65	640	5120	Two-dose	70	480	2560

*DPV* day post vaccination (first dose), *DPC* day post challenge. Pigs were challenged on 35 DPV

(−/−): Control pigs without challenge; (−/+): Control pigs challenged with CSFV

One-dose: Pigs vaccinated with one dose KNB-E2 and then challenged with CSFV

Two-dose: Pigs vaccinated with KNB-E2 twice and then challenged with CSFV

## Discussion

Commercial E2 subunit vaccines previously available in Europe were marketed as two-dose vaccines for basic vaccination for homologous protection. Here we present data showing that vaccination with one dose KNB-E2 protects pigs from subgenotype heterologous (1.1 vs. 1.3) CSFV challenge. Pigs vaccinated with KNB-E2 did not have any fever or growth retardation after CSFV challenge. CSFV was not detected in the blood, nasal cavity, lung, kidney, lymph node, and tonsil in vaccinated pigs 15 days post challenge. Our report demonstrates that subunit vaccine KNB-E2 is safe and effective at stimulating immunity against heterologous and geographically (Central-South America) relevant CSFV in an experimental setting in the U.S.

It has been well established that CSFV E2 protein is the major protective antigen and can elicit neutralizing antibody, thus it is frequently used as the antigen for subunit vaccine development (see reviews [6, 9, 28]). Because attenuated CSFV vaccine HCLV strain is well-known for its efficacy and safety, the HCLV E2 gene (genotype 1.1) was cloned and expressed with the insect cell/baculovirus system in this study. Purified E2 proteins mainly present as homodimers, which is identical to the native glycosylated E2 protein reported in previous studies [21, 29]. Furthermore, we found that E2 homodimers have higher affinity to E2-specific mAb WH211 than do the monomers, indicating that oligomerization and glycosylation of E2 protein are important for the induction of protective immune response and neutralizing antibodies.

The efficacy of the two commercial E2 subunit vaccines has been extensively evaluated in various vaccination-challenge studies in pigs. The E2 antigen in *BAYOVAC CSF E2* was originated from CSF Brescia [30], a genotype 1.2. Although a single vaccination with this vaccine can significantly reduce mortality of pigs challenged with homologous CSFV up to 13 months after a single vaccination [13], it did not protect pigs from developing fever [11]. Different from *BAYOVAC CSF E2*, the E2 antigen in *Porcilis Pesti* was originated from CSFV Alfort/Tubingen [28], a genotype 2.3. It was reported that single vaccination with *Porcilis Pesti* failed to protect immunized pigs and prevent horizontal transmission of CSFV (Alfort/187, genotype 1.1) to unvaccinated sentinel pigs [31]. Others have shown that *Porcilis Pesti* can totally prevent horizontal transmission 14 DPV and significantly reduce transmission 7 DPV [10].

In contrast, pigs immunized with one dose KNB-E2 are protected from a subgenotype heterologous CSFV challenge without the development of fever and growth retardation, and CSFV was cleared from the pigs by 15 DPC. The differential efficacy between KNB-E2 and the commercial E2 subunit vaccines may be due to the fact that we use E2 protein from genotype 1.1 C-strain CSFV and/or the E2 antigens in KNB-E2 are in the native dimeric conformation. This speculation is consistent with the report from others showing that sows vaccinated with E2 proteins from CSFV genotype 1.2 were better protected against clinical CSF than sows vaccinated with E2 proteins from CSFV genotype 2.3 when these pigs were challenged with CSFV genotype 2.1 [32]. Alternatively, the higher (75 μg/dose for KNB-E2 vs. 32 μg/dose for BAYORVAC CSF E2) amount of E2 protein used in our vaccine may also contribute to the superior efficacy after one dose immunization.

In addition, we have also found that pigs immunized with one dose or two doses of KNB-E2 have different antibody responses to CSFV challenge. Prior to challenge, the VNA titer and E2-specific antibody in pigs from the One-dose group were lower than that from pigs in the Two-dose group (Table 3 and Fig. 4). However, pigs in the One-dose group generated higher anti-CSFV neutralizing antibody and E2-specific antibody after challenge than that of pigs in the Two-dose group. It seems that CSFV challenge with Honduras/1997 may function as a booster vaccination with live viruses, which resulted in the induction of a stronger immunological response and a larger amounts of neutralizing antibody than does a boost vaccination with a subunit vaccine. We speculate that the high levels of VNA and E2-specific antibodies prior to challenge in the Two-dose pigs may accelerate the inactivation and removal of circulating CSFV post challenge, which may lead to fewer or no live CSFV available for further immunological stimulation.

The dynamics of anti-CSFV neutralizing antibody produced in the vaccinated groups after challenge is consistent with the changes of white blood cells. As shown in Figs. 3 and 4, the trends (decrease or increase) of WBC and lymphocytes parallel the trends of E2-specific antibody levels and VNA titers in vaccinated pigs compared with that in control pigs. Interestingly, the impact of KNB-E2 vaccination on leukocyte homeostasis is different from that of MLV C-strain vaccine, as evidenced by the fact that dramatic increase of leukocyte cells in pigs vaccinated with KNB-E2 was not observed in pigs immunized with the C-strain vaccine [33]. The meaning of this difference has yet to be explored in future studies.

In contrast to all vaccinated pigs, control pigs challenged with CSFV developed significant levels of anti-E^rns^ antibody at 15 days post challenge. This observation indicates that KNB-E2 has the DIVA characteristic as a CSF vaccine. However, we do recognize that although the challenge CSFV strain Honduras/1997 used in our study is from the Americas and heterologous to the origin strain of the E2 gene, it is not a recent outbreak strain. Our study design was limited by the current availably of CSFV in an academic setting in the U.S. – a CSF-free country. Because most of the presently circulating strains in the world belong to genotype 2, future studies are planned to better understand the protective ability of KNB-E2 against the CSFV in the field.

## Conclusion

CSFV E2 protein was successfully generated by insect cell/baculovirus expression system and the purified E2 protein was formulated with an oil-in-water emulsion adjuvant as a CSF subunit vaccine (KNB-E2) for pigs. Pigs immunized with one dose KNB-E2 can be clinically protected from CSFV challenge. This novel subunit vaccine has DIVA characteristics, can be manufactured in CSF-free regions, and is suitable for CSF prevention and control in both CSF endemic area and emergency outbreaks.

## Abbreviations

CSF: Classical swine fever
MLV: Modified live virus
CSFV: Classical swine fever virus
TCID_50_: 50 % tissue culture infective dose
DIVA: Differentiation of infected from vaccinated animals
HCLV: Hog cholera lapinised virus
WBC: White blood cells
PBS: Phosphate-buffered saline
DPV: Days post vaccination
DPC: Days post challenge
ELISA: Enzyme-linked immunosorbent assay
VNA: Virus neutralizing antibody
β-ME: β-mercaptoethanol
